# Mooring line snap-back trauma leading to bilateral lower limbs amputation: a case exploration with preventive strategies

**DOI:** 10.1093/jscr/rjaf181

**Published:** 2025-04-22

**Authors:** Wensai Ji, Hongzhang Liu, Di Wu, Qiuhuan Xu, Junyi Zhang, Jiayan Chen, Shiyu Han, Qing Sun

**Affiliations:** Mental Health Center, Qingdao University, No. 308 Ningxia Rd, Qingdao 266071, China; Department of Medical Affairs, No. 971 Hospital of the PLA Navy, No. 22 Minjiang Rd, Shinan Dist., Qingdao 266071, China; Department of Hand Surgery, No. 971 Hospital of the PLA Navy, No. 22 Minjiang Rd. Shinan Dist., Qingdao 266071, China; Department of Military and Special Medicine, No. 971 Hospital of the PLA Navy, No. 22 Minjiang Rd, Shinan Dist., Qingdao 266071, China; Department of Urology, No. 971 Hospital of the PLA Navy, No. 22 Minjiang Rd, Shinan Dist., Qingdao 266071, China; Department of Medical Affairs, No. 971 Hospital of the PLA Navy, No. 22 Minjiang Rd, Shinan Dist., Qingdao 266071, China; Department of Military and Special Medicine, No. 971 Hospital of the PLA Navy, No. 22 Minjiang Rd, Shinan Dist., Qingdao 266071, China; Department of Medical Affairs, No. 971 Hospital of the PLA Navy, No. 22 Minjiang Rd, Shinan Dist., Qingdao 266071, China; Department of Military and Special Medicine, No. 971 Hospital of the PLA Navy, No. 22 Minjiang Rd, Shinan Dist., Qingdao 266071, China

**Keywords:** bulk carrier, snap back trauma, mooring accident, limb amputation

## Abstract

The report focuses on mooring operations-related accidents, especially those involving mooring line partings for bulk carriers. It presents a case of a young seafarer who suffered severe injuries from a snap-back mooring line during disembarking. The causes of the accident, including factors like abnormal fendering compression and meteorological/hydrological conditions, are analyzed. Moreover, it discusses prevention measures for such accidents, treatment strategies for snap-back trauma which is a complex and multidisciplinary process, and key aspects regarding the long-term prognosis and quality of life of the cases. The study emphasizes the importance of establishing comprehensive plans and training programs due to the common occurrence of such accidents and their potentially severe consequences, as there's a lack of systematic reviews on on-site emergency care strategies in general.

## Introduction

Mooring operations are critical marine activities involving the securing of a ship to a dock using lines [1]. For bulk carriers, these operations can be particularly dangerous due to various factors [2]. According to the UK Protection and Indemnity Club, accidents during mooring operations “are the 7th most frequent cause of personal injuries” [3], and the impairments concerning ropes/lines that parted while handling them should be held accountable for ⁓53% of the total mooring accidents [4]. Therefore, it is obvious that the severity should be a general concern for offshore shipping management personnel [5].

Snap-back is the sudden release of the energy stored in a tensioned mooring line when it parts as it reverts to its original length. When a synthetic mooring line breaks, the snap-back effect can be extremely powerful and may result in crushing and tearing of soft tissues, bone fracture, and even death [6–8].

This study reported a seafarer in which a large bulk carrier experienced a sudden parting of a spring line during mooring at the dock due to changes in weather and hydrological conditions. Despite enough training, the victim sustained severe injuries requiring bilateral lower limb amputation, highlighting the need for multidisciplinary management and strict adherence to safety protocols to prevent such traumas.

## Case report

On 1 November 2024 (D0), while disembarking from the bulk carrier moored at the dock, this 22-year-old male seafarer heard a great noise and was immediately hit below the knee by a spring line that abruptly parted, resulting in severe traumatic hemorrhage and complete mutilation of both calves. The on-site partner crews swiftly used their own belts as tourniquets to perform basic dressing aid and called for assistance from the bulk duty officer. The case was then conscious, in severe pain, and in a state of severe anxiety and agitation. He was admitted to the department of hand and microsurgery of a local tertiary hospital 2 h after the injury, while on transportation, he was administered an appropriate dose of morphine and a vascular access was established, with 1 L of sodium lactate Ringer’s solution infused for fluid resuscitation.

**Figure 1:**
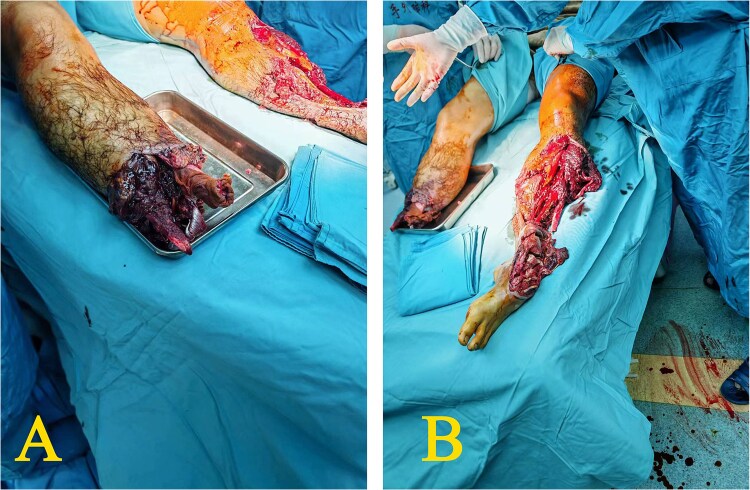
Condition of both calves when arriving at the emergency room (D0) (A) the right calf: Completely severed with tendon retraction and exposure of the proximal tibia and fibula; moderate contamination of the wound; (B) the left calf: Completely severed below the mid-shaft, leaving only 3 cm of skin, accompanied by significant distal tissue destruction characterized by comminuted fractures of the left tibia, calcaneus, and talus.

Upon admission, the emergency department witnessed that case’s right calf was completely severed, showing tendon retraction and exposure of the proximal tibia and fibula, with localized ischemic necrosis and moderate contamination of the wound (Fig. 1A). The left calf was also completely severed below the mid-shaft, leaving only 3 cm of skin, accompanied by significant distal tissue destruction characterized by comminuted fractures of the left tibia, calcaneus, and talus. The wound was moderately contaminated with extensive soft tissue damages (Fig. 1B).

**Figure 2:**
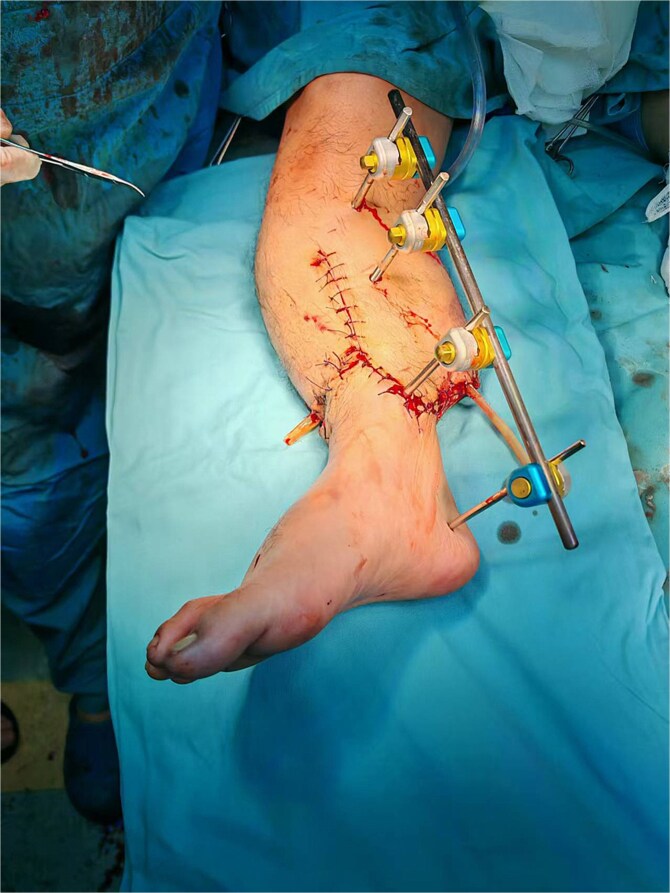
The right calf after debridement, shortened artery repair with replantation and external fixation of the tibia (D0).

Following adequate blood transfusions and fluid resuscitation, the case underwent a series of procedures including debridement, shortened artery repair with replantation of the right calf, external fixation of the right tibia (Fig. 2), and underwent amputation of the left calf. Postoperatively, the case’s general condition was stable; however, there was considerable wound seepage, and healing was progressing slowly.

**Figure 3:**
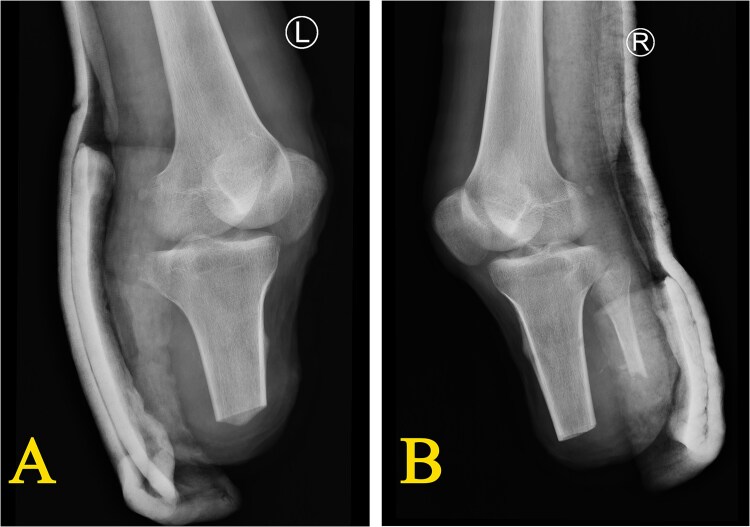
X-ray after bilateral lower limb amputation (D34) (A) post-operative condition of the left calf after local flap transfer repair, showing near complete healing; (B) post-operative condition of the right calf after debridement with vacuum sealing drainage and application of a cast for external fixation.

On D10, the case experienced a sudden massive bleeding from the surgical site of the right calf, leading to an inevitable emergency amputation of the right lower leg. On D34, under subarachnoid anesthesia, he underwent further debridement and suturing with vacuum sealing drainage, local flap transfer repair of the left calf, adhesiolysis of the knee joint, and application of a cast for external fixation (Fig. 3A and B). The case was then transferred to the rehabilitation department for psychological support and prosthesis fitting evaluation.

## Discussion

### Accidents prevention

Although the parting of mooring lines on large ships is rare, mooring operations for large bulk carriers are generally considered highly risky [9]. While factors such as estimated line strength and limited service life are not the primary considerations in emergency safety assurances, from a clinical and forensic medical perspective, the potential for snap-back trauma associated with line parting presents new challenges for shore-based medical personnel and on-site emergency responders [10].

The incident in this case, influenced by changes in weather, hydrology, and mooring techniques, highlights the unpredictability of such accidents, particularly when staff are trained properly yet still encounter unforeseen circumstances. The implementation of robust safety protocols and the strategic marking of snap-back zones must be continually reviewed, as emerging evidence suggests that current practices may inadvertently elevate risks.

### Treatment strategies for snap-back trauma

**Figure 4:**
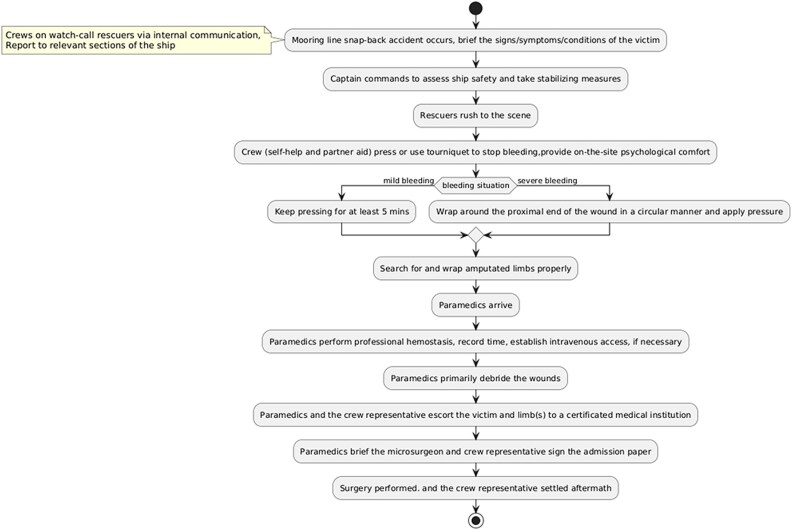
The emergency response and strategy flowchart tailored for large bulk carriers.

Managing injuries resulting from mooring line snap-back necessitates a multidisciplinary approach. Recent advancements in surgical techniques focus on optimizing amputation sites to facilitate enhanced wound healing and prosthetic integration. In the reported case, although the left calf was ultimately amputated, surgical efforts to reattach the right calf aimed at promoting functional recovery and improving outcomes. Based on the treatment experience from this case, we created an emergency response strategy flowchart for large bulk carriers (Fig. 4).

Post-surgical rehabilitation plays a vital role and should encompass early mobilization, physical therapy to enhance muscle strength and joint function, and occupational therapy to support patients in regaining independence. Furthermore, significant progress in prosthetic technology provides newer, lighter, and functionally superior devices that contribute positively to post-trauma rehabilitation.

### Long-term prognosis and quality of life

Upon discharge, the patient was to receive a prosthesis, with specific considerations addressed regarding recovery and overall quality of life. Postoperative care must emphasize thorough monitoring of the residual limb to prevent complications, including pressure ulcers. Education on proper positioning to minimize friction and pressure, along with professional guidance in shaping the residual limb for optimal prosthetic fitting, is crucial. Rehabilitation must start with basic isometric exercises, gradually increasing resistance to build muscle strength for prosthetic use. Psychological support is imperative, particularly for younger individuals adapting to life changes following limb loss, and efforts should be made to engage patients in support networks that enhance their confidence and aid in emotional adjustment. Preventative strategies for complications such as phantom limb pain and residual limb joint contractures should be implemented, employing a combination of medication and physical therapy to preserve joint functionality.

## Conclusion

Despite efforts from emergency personnel, the case underwent amputation and faced significant distress. This highlights the need for specialists to advocate for comprehensive training programs and strategies to manage such injuries effectively.
